# **L**-Ascorbic Acid: A Multifunctional Molecule Supporting Plant Growth and Development

**DOI:** 10.1155/2013/795964

**Published:** 2013-01-17

**Authors:** Daniel R. Gallie

**Affiliations:** Department of Biochemistry, University of California, Riverside, CA 92521-0129, USA

## Abstract

L-Ascorbic acid (vitamin C) is as essential to plants as it is to animals. Ascorbic acid functions as a major redox buffer and as a cofactor for enzymes involved in regulating photosynthesis, hormone biosynthesis, and regenerating other antioxidants. Ascorbic acid regulates cell division and growth and is involved in signal transduction. In contrast to the single pathway responsible for ascorbic acid biosynthesis in animals, plants use multiple pathways to synthesize ascorbic acid, perhaps reflecting the importance of this molecule to plant health. Given the importance of ascorbic acid to human nutrition, several technologies have been developed to increase the ascorbic acid content of plants through the manipulation of biosynthetic or recycling pathways. This paper provides an overview of these approaches as well as the consequences that changes in ascorbic acid content have on plant growth and function. Discussed is the capacity of plants to tolerate changes in ascorbic acid content. The many functions that ascorbic acid serves in plants, however, will require highly targeted approaches to improve their nutritional quality without compromising their health.

## 1. Introduction

Vitamin C (l-ascorbic acid) is a water-soluble antioxidant that serves a predominantly protective role. Despite the fact that most mammals can synthesize ascorbate (Asc), humans (along with other primates, bats, and guinea pigs) are unable to make vitamin C as a result of a mutation to the gene encoding l-gulono-1,4-lactone oxidase, the last enzyme in the Asc biosynthetic pathway [1]. Although the symptoms associated with severe vitamin C deficiency, for example, weak joints, bleeding gums, and skin discoloration due to ruptured blood vessels, had been observed and described as early as 1497 by Vasco da Gama among his crew during their voyage around the southern tip of Africa to India, it was not until 1747 that James Lind demonstrated that consumption of citrus fruit prevented or cured the disorders associated with scurvy. Because of this, vitamin C was originally referred to as the “antiscorbutic factor.” Vitamin C (l-threo-hex-2-enono-1,4-lactone) was eventually isolated in 1928 by Dr. Szent-Gyorgyi although he was uncertain of its function. Only in 1932 did crystallization of the physiologically active compound isolated from natural sources identify the antiscorbutic factor as vitamin C [2–4], and its structure determined the following year [5].

In animals, ascorbate is involved in the synthesis of carnitine and collagen, an important component of skin, scar tissue, tendons, ligaments, and blood vessels [6–8]. As a result, Asc is essential for the repair and maintenance of cartilage, bones, and teeth and during wound healing. It is also important in increasing the absorption of nonheme iron from plant-based foods. The National Academy of Sciences has established 90 mg/day (for adult males) and 75 mg/day (for adult females) as the Recommended Dietary Allowance (RDA) for vitamin C [9]. In USA, 20–30% of adults get less than 60 mg of vitamin C from dietary sources, and the extent of subclinical vitamin C deficiency in the population has not been appreciated [10].

As plant-based foods constitute the principle source of vitamin C in human diets, the possibility of increasing the Asc content of plants to improve their nutritive value has received considerable attention in recent years [11–13]. Asc, however, serves many functions in plants. For example, it is a major redox buffer [14] and serves as a required cofactor for several enzymes and as a major antioxidant [15, 16]. Asc also regulates cell division and growth [17] and is involved in signal transduction [14, 18]. Although plants can tolerate moderate changes to the endogenous level of Asc, such alterations are not without consequences as might be expected for a molecule so inextricably linked to plant growth and health. This paper will examine and evaluate the approaches that have been used to increase Asc content in plants including those that have focused on increasing Asc biosynthesis as well as those that have targeted the efficiency of Asc recycling. The consequences of altering Asc levels on plant growth and development, health, and their ability to respond to environmental stress will also be presented.

## 2. Biosynthesis of **l**-Ascorbic Acid in Plants

**Figure 1:**
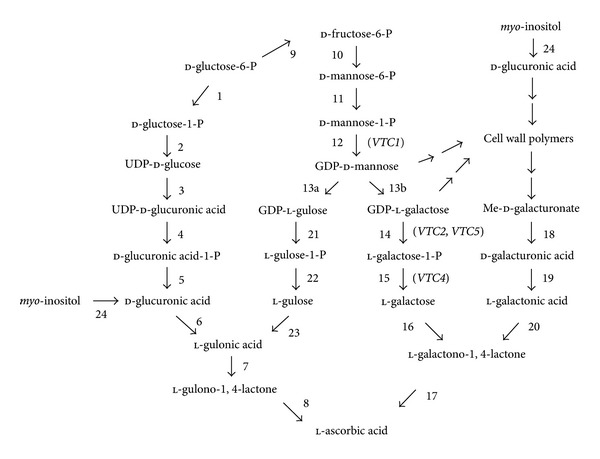
l-Ascorbic acid biosynthetic pathways in plants and animals. Reactions 1–8 represent the pathway in animals and reactions 9–24 represent the pathways in plants. Enzymes in each pathway are 1, phosphoglucomutase; 2, UDP-glucose pyrophosphorylase; 3, UDP-glucose dehydrogenase; 4, glucuronate-1-phosphate uridylyltransferase; 5, glucuronate 1-kinase; 6, glucuronate reductase; 7, aldonolactonase (aka. gluconolactonase); 8, gulono-1,4-lactone oxidase or dehydrogenase; 9, glucose-6-phosphate isomerase; 10, mannose-6-phosphate isomerase; 11, phosphomannose mutase; 12, GDP-mannose pyrophosphorylase (mannose-1-phosphate guanylyltransferase) (*VTC1*); 13, GDP-mannose-3′, 5′-epimerase; 14, GDP-l-galactose phosphorylase (*VTC2* and *VTC5*); 15, l-galactose-1-phosphate phosphatase (*VTC4*); 16, l-galactose dehydrogenase; 17, l-galactono-1,4-lactone dehydrogenase; 18, methylesterase; 19, d-galacturonate reductase; 20, aldonolactonase; 21, phosphodiesterase; 22, sugar phosphatase; 23, l-gulose dehydrogenase; 24, *myo*-inositol oxygenase. Adapted from Agius et al. [35].

In mammals, d-glucuronic acid is generated from d-glucose via the intermediates: d-glucose-1-P, UDP-glucose, UDP-d-glucuronic acid, UDP-d-glucuronic acid-1-P, and d-glucuronic acid (Figure 1). d-Glucuronic acid is then converted to l-gulonic acid by glucuronate reductase which is then converted to gulono-1,4-lactone by aldono-lactonase (aka. gluconolactonase) [19]. l-Ascorbic acid is generated from gulono-1,4-lactone through the action of gulono-1,4-lactone oxidase which produces 2-keto-gulono-*γ*-lactone which spontaneously converts to l-ascorbic acid. The initial elucidation of the Asc biosynthetic pathway in plants suggested that it differed substantially from the animal pathway. The Smirnoff-Wheeler pathway in plants involves the generation of l-ascorbic acid from l-galactose [20] (Figure 1). l-Galactose is generated from mannose-1-phosphate by the conversion of guanosine diphosphate (GDP)-mannose to GDP-l-galactose by GDP-mannose-3′, 5′-epimerase [21] which is then converted to l-galactose. l-Galactono-1,4-lactone is synthesized from the oxidation of l-galactose by the NAD-dependent l-galactose dehydrogenase. l-Galactono-1,4-lactone serves as the immediate precursor of l-ascorbic acid and is oxidized to l-ascorbic acid by l-galactono-1,4-lactone dehydrogenase which is located on the outer side of the inner membrane of mitochondria [22, 23]. Although the initial steps of the pathway are located in the cytosol, the oxidation of l-galactono-1,4-lactone via cytochrome c in the mitochondria suggests the integration of Asc biosynthesis with energy metabolism and the cellular redox state. Feeding experiments with leaf tissue demonstrated that l-galactose and l-galactono-1,4-lactone are indeed converted to l-ascorbic acid and can rapidly increase the Asc pool size [20, 24, 25]. Several mutants that displayed various degrees of Asc deficiency have been isolated in Arabidopsis and described as *vtc* mutants. The *vtc1* mutant results from a mutation in GDP-mannose pyrophosphorylase, the *vtc2* and *vtc5* mutants result from a mutation in GDP-l-galactose phosphorylase (or GDP-l–galactose-hexose-1-phosphate guanyltransferase), and the *vtc4* mutant results from a mutation in l-galactose-1-P phosphatase, all enzymes in the Smirnoff-Wheeler pathway [26–30] (Figure 1).

In contrast to the single mammalian biosynthetic pathway, however, evidence for additional Asc biosynthetic pathways in plants has accumulated in recent years. A second Asc biosynthetic pathway was suggested by the radiotracer work of Loewus and Kelly [31] using detached ripening strawberry fruit in which d-galacturonic acid-1-^14^C was metabolized to l-ascorbic acid-6-^14^C by an inversion pathway. This suggested a pathway in which d-galacturonic acid, generated from the breakdown of pectin in the ripening fruit, is reduced to l-galactonic acid through the action of an NADPH-dependent d-galacturonic acid reductase (GalUR), and the l-galactonic acid then spontaneously converts to l-galactono-1,4 lactone [32] (Figure 1). As in the l-galactose pathway described above, l-galactono-1,4-lactone dehydrogenase converts l-galactono-1,4 lactone to l-ascorbic acid. The radiotracer data in strawberry fruits and the observation that feeding of a methyl ester of d-galacturonic acid to cress seedlings and Arabidopsis cell cultures lead to a significant increase in l-ascorbic acid [33, 34] indicated the presence of GalUR. Overexpression of GalUR from strawberry in Arabidopsis increased whole-plant Asc content 2- to 3-fold (Table 1) [35]. Additional radiotracer data indicated, however, that the generation of l-ascorbic acid from GalUR could account for only a small portion of the total ascorbate produced in strawberry fruit [36]. This suggested that this pathway may make only a minor contribution to Asc biosynthesis or may be specific to certain organs under specific conditions.

A link with the Asc biosynthetic pathway in animals has been suggested in plants from studies with GDP-mannose 3′, 5′-epimerase. This enzyme not only catalyzes the conversion of GDP-d-mannose to GDP-l-galactose in the l-galactose pathway [21] but can also generate GDP-l-gulose from the 5′-epimerization of GDP-d-mannose [40] (Figure 1). Whether GDP-l-galactose or GDP-l-gulose is produced by GDP-mannose 3′, 5′-epimerase from GDP-d-mannose appears to be dependant on the molecular form of the enzyme [40]. Although the remaining steps in this pathway have yet to be demonstrated, l-gulonic acid and l-gulono-1,4-lactone dehydrogenase activity are present in plants [40, 41]. Further evidence for the mammalian biosynthetic pathway in plants was provided by the expression of l-gulono-1,4-lactone oxidase from rat in lettuce and tobacco which increased Asc 4- to 7-fold (Table 1) [38]. Expression from the same gene reversed the foliar Asc deficiency of the Arabidopsis *vtc* mutants, restoring Asc content to a level that was similar or greater than in wild-type plants [42]. This supports the notion that l-gulono-1,4-lactone is present in plants. The possibility that l-galactono-1,4-lactone might have served as a substrate for the rat l-gulono-1,4-lactone oxidase probably cannot account for the increase in the Asc pool size as the level of l-galactono-1,4-lactone is reduced in the *vtc1* mutant. To what extent this alternative pathway may be operative in plants is unknown. As with other approaches to overexpress enzymes that are not normally present in plants or whose expression may be subject to organ-specific regulation, ectopic overexpression from a gene can cause the ectopic expression of a pathway or the introduction of a novel pathway in plants.

d-Glucuronic acid, an intermediate of the mammalian biosynthetic pathway, can be generated in plants by *myo*-inositol oxygenase (Figure 1). Ectopic expression of an Arabidopsis gene having homology to *myo*-inositol oxygenase from pig increased Asc levels in Arabidopsis [39]. Once again, the extent to which this pathway may be operative in plants is unknown although radiotracer data indicates that *myo*-inositol does not function as a precursor of l-ascorbic acid in ripening strawberry fruit or in parsley leaves [36]. Whether there are additional pathways for the biosynthesis of l-ascorbic acid in plants remains unknown at this point. However, as the Asc content in the* vtc2* mutants is just 10–20% of the level in wild-type Arabidopsis and *vtc2*/*vtc5* double mutants bleach within one week of transfer to l-Gal-free medium [28], the Smirnoff-Wheeler pathway is likely responsible for the bulk of foliar Asc biosynthesis in Arabidopsis and perhaps in other plant species as the other pathways are unable to compensate for the loss in Asc biosynthetic capacity in these mutants.

## 3. l-Ascorbic Acid Transport in Plants

### 3.1. Intracellular Transport of Ascorbic Acid

Because Asc is transported throughout a plant, changes in Asc content in one part of a cell or tissue may affect its levels in other cellular compartments or tissues. Consequently, attempts to alter Asc content need to consider Asc transport mechanisms present in plants. Despite the fact that the last step in the Asc biosynthetic pathway takes place on the inner membrane of mitochondria [22, 43], Asc is found throughout the cell including the apoplast. Consequently, Asc must be transported to all other compartments of the cell in which it is present [44, 45]. Given that the bulk of Asc would exist in its negatively charged form at physiological pH values (p*K*_*a*1_ = 4.2; p*K*_*a*2_ = 11.6), diffusion of Asc through lipid bilayers is unlikely. Although uncharged, dehydroascorbate (DHA), the oxidized form of Asc, is not hydrophobic enough for it to diffuse across cellular membranes. In animal cells, transport of DHA but not Asc utilizes glucose transporters [46–48]. Evidence indicates that transport of Asc and DHA into plants cells occurs through energized uptake at the expense of the transmembrane proton motive force and that the energized transport of Asc was calculated to be approximately 650 nmol m^−2^ leaf area s^−1^ [49]. Due to the presence of dehydroascorbate reductase (DHAR), which catalyzes the reduction of DHA to Asc, the latter predominates in the cytosol. The absence of DHAR in the apoplast, however, means that once Asc is transported out of the cell, it undergoes oxidation to DHA. This results in DHA being the predominant form in the apoplast from which it is efficiently transported back into the cytosol for reduction to Asc.

Both Asc and DHA are taken up by plasma membrane vesicles derived from *Phaseolus vulgaris* and in protoplasts from tobacco and barley but with a demonstrated higher affinity for DHA than for Asc [50–54]. Although Asc transporters have yet to be identified definitively, analysis of the *Arabidopsis thaliana* genome identified twelve genes sharing similarity with known nucleobase-ascorbate transporters (NATs) from other species [55], suggesting the possibility that these genes encode putative ascorbate transporters. Localization to the plasma membrane was observed for three of the AtNAT family members. Single knockout mutants of all *AtNAT* genes, as well as some double and triple mutants, did not exhibit any obvious phenotype, indicating functional redundancy among the members of this gene family [55]. The elucidation of the function of AtNAT proteins and whether they are actually involved in Asc transport remains to be determined.

Asc uptake in chloroplasts employs a specific transporter [56, 57]. Analysis of mitochondria from tobacco indicated that the uptake of DHA and glucose occurs by facilitated diffusion and is mediated by the same transporter [58]. In contrast, uptake of Asc in mitochondria occurs with low affinity and therefore likely crosses the mitochondrial membrane in its oxidized form [58].

### 3.2. Long-Distance Transport of Ascorbic Acid 

The observation that radiolabeled Asc applied to leaves accumulated in the phloem and is transported to root tips, shoots, and floral organs, but not to mature leaves, was the first demonstration of phloem-mediated, long-distance transport of Asc [59]. Feeding l-galactono-1,4-lactone, the precursor to Asc, to intact leaves of Arabidopsis or *Medicago sativa *resulted in up to an 8-fold increase in Asc content in the treated leaf and up to a 3-fold increase of Asc in sink tissues [59]. Moreover, the increase in Asc was proportional to the amount of the Asc precursor applied [59]. In potato, feeding l-galactono-1,4-lactone or l-galactose to source leaves resulted in a substantial increase in Asc in phloem exudates as well as in sink organs, such as flowers and developing tubers [60]. Foliar Asc content was 2-fold higher during the day than at night which was reflected in phloem exudates, suggesting that Asc content in the phloem is highly responsive to changes in Asc biosynthesis in source leaves [60]. Because the enzymes needed for Asc biosynthesis are present in the plant phloem [61], the relative contributions of its transport from source leaves versus its *in situ* biosynthesis in the phloem on the accumulation of Asc in sink tissues remains to be determined. The observation that there is a 3- to 10-fold higher biosynthetic capacity and a lower turnover rate for Asc in mature leaves than in sink tissues [59] underscores the importance of phloem-mediated transport of Asc during the growth and development of sink organs.

Asc feeding to leaves of Micro-Tom tomato plants demonstrated translocation and accumulation of Asc in immature green fruits [62]. The same feeding experiments demonstrated a decrease in Asc translocation with subsequent ripening. l-Galactose feeding (representing the d-mannose/l-galactose pathway) also increased the Asc content in immature green fruit, but feeding with either d-galacturonate or l-gulono-1,4-lactone did not [62]. In red ripened fruits, however, feeding with l-galactose (representing the d-mannose/l-galactose pathway) or d-galacturonate (representing the D-galacturonate pathway) increased the Asc content of the fruits whereas feeding with l-gulono-1,4-lactone (representing the *myo*-inositol/d-glucuronate or l-gulose pathways) did not [62]. Correlating with the feeding results was the detection of the activities for the last two enzymes, that is, d-galacturonate reductase and aldonolactonase, of the d-galacturonate pathway in immature and ripe fruits [62]. These data demonstrate Asc translocation from source leaves and they suggest that a switch from the d-mannose/l-galactose pathway as the sole means for Asc synthesis during early fruit development to a combination of this pathway and the alternative d-galacturonate pathway occurs during fruit ripening.

## 4. Role of l-Ascorbic Acid in Plants

### 4.1. Ascorbic Acid as an Enzyme Cofactor

Asc can serve as an enzyme cofactor, for example, for violaxanthin de-epoxidase (VDE) [63], which catalyzes the conversion of violaxanthin to zeaxanthin as part of the xanthophyll cycle. Zeaxanthin, and thus the correct functioning of the xanthophyll cycle, is required for the energy-dependent, thermal dissipation of excess absorbed excitation energy during nonphotochemical quenching (NPQ). Asc also participates in the regeneration of *α*-tocopherol (vitamin E) from the tocopheroxyl radical [64]. In addition, Asc functions as a cofactor for enzymes such as prolyl and lysyl hydroylases [65, 66]; 1-aminocyclopropane-1-carboxylate oxidase that catalyzes the last reaction of ethylene biosynthesis [63, 67, 68]; 2-oxoacid-dependent dioxygenases including those involved in the synthesis of abscisic acid [69], gibberellic acid [70–73], and anthocyanins [74–76]. Asc is also involved in the regulation of cell elongation and progression through the cell cycle [45, 77].

### 4.2. Ascorbic Acid Detoxifies Reactive Oxygen Species Generated during Photosynthesis

Asc is just one of many antioxidants present in plants that include glutathione (GSH), carotenes, carotenoids, tocopherols, and polyphenols. Asc is abundant in leaf tissue and is present in millimolar concentrations in the chloroplast stroma [78, 79]. As the concentration of Asc exceeds that of other antioxidants, it is considered the major antioxidant present in plants. Because of its high concentration, Asc serves as the major contributor to the cellular redox state and is important in maintaining photosynthetic function [64, 80]. Asc is used to detoxify reactive oxygen species (ROS), for example, singlet oxygen (^1^O_2_), superoxide anion (O_2_^∙−^), hydroxyl radical (OH^∙^), and hydrogen peroxide (H_2_O_2_), in order to protect the photosynthetic apparatus and other cellular components from oxidative damage [81]. ROS are detoxified through the action of antioxidants such as Asc and GSH either directly or in reactions catalyzed by superoxide dismutase (SOD), ascorbate peroxidase (APX), and catalase (CAT) [82, 83]. The ascorbate-glutathione (Asc-GSH) cycle, which includes the activities of monodehydroascorbate reductase (MDAR), dehydroascorbate reductase (DHAR), and glutathione reductase (GR), plays an important recycling role to regenerate Asc and GSH when they undergo oxidation through their reaction with ROS during conditions of oxidative stress [84].

Asc can serve as a direct electron donor to photosystem I (PSI) and photosystem II (PSII) in isolated thylakoids under conditions where the water-oxidase complex is impaired, for example, under high light stress [85, 86], although *in planta* confirmation is needed. Photoreduction of monodehydroascorbate (MDHA), produced following the oxidization of Asc, functions to maintain electron transport flow when NAPD^+^ is limiting by competing with Fd-NAPD^+^ for electrons at the reducing side of PSI [87, 88]. Asc is also used by APX to convert H_2_O_2_ to water, and Asc can directly scavenge other ROS that are produced during aerobic metabolic processes such as photosynthesis or respiration [81] although the extent to which the direct reduction of ROS occurs *in planta* remains to be determined.

Despite its essential role in supporting life, oxygen can be highly damaging. In the chloroplast, excess light, can result in the production of O_2_^∙−^ which is then converted by SOD to H_2_O_2_ and further reduced to H_2_O by APX in the water-water cycle [89]. This cycle serves to maintain electron flow through the photosystems. Exposure to many abiotic stresses, including cold, drought, or high light can exacerbate ROS production by creating conditions of light stress at lower photon flux density. H_2_O_2_ inactivates APX within seconds if Asc recycling is impaired [90]. H_2_O_2_ can also inhibit CO_2_ assimilation by inhibiting several Calvin cycle enzymes [91]. H_2_O_2_ can also be generated in response to exposure to pollutants such as ozone [92, 93]. As H_2_O_2_ serves as a signaling intermediate in guard cells which promotes stomatal closure, plants attempt to limit further exposure to ozone by closing their stomata which also limits photosynthetic activity [94, 95]. Despite this defense mechanism, an acute exposure to ozone can result in damage to cell membranes or even induce programmed cell death [96–98].

Although most plants grow photoautotrophically, they often must cope with rapid changes in the level of incident light resulting from the angle of the sun, changes in cloud cover, or from shade produced by neighboring plants. Despite the conversion of solar energy into chemical energy, the capacity of photosynthesis to use absorbed light energy is limited. Excess light energy can be dangerous as it can result in the production of triplet state chlorophyll (^3^Chl) that can transfer energy to ground-state O_2_ to produce singlet oxygen (^1^O_2_). In addition, the overreduction of the photosystems can result in the generation of other ROS such as O_2_^∙−^ and H_2_O_2_ [81]. Such ROS can damage the protein subunits, membranes, and pigments of PSI and PSII, resulting in protein degradation, inactivation of reaction centers, and inhibition of the subsequent repair mechanisms of the reaction center [99, 100].

The importance of Asc in protecting photosynthetic function has been shown with *vtc *mutants of Arabidopsis [27, 101]. The *vtc1* mutant, defective in GDP-mannose pyrophosphorylase, accumulates 25%–30% of the wild-type level of Asc. *vtc1* plants experience chronic photooxidative stress in high light and are hypersensitive to ozone, sulfur dioxide, or UVB light [25, 27, 102]. However, *vtc1* plants exhibit only a moderately slower growth rate under normal growth conditions [102], suggesting that much of the Asc in plants is essential to respond to conditions of oxidative stress. The *vtc2* mutant, defective in GDP-l-galactose phosphorylase, contains about 10–20% of wild-type Asc and exhibits sensitivity to ozone [101]. The *vtc2* mutant also has reduced energy-dependent NPQ (qE), in which excess absorbed light energy is thermally dissipated [103]. As Asc is a cofactor for the VDE- catalyzed deepoxidation of violaxanthin and antheraxanthin to zeaxanthin, the latter of which contributes to energy-dependent NPQ, the reduction in Asc content in the *vtc2* mutant limits VDE activity [104]. The *vtc2* mutant also experiences increased photoinhibition when transferred from low to high light which is accompanied by increased lipid peroxidation suggesting a higher level of photooxidative damage [102, 103, 105].

*VTC2* and* VTC5* encode GDP-l-galactose phosphorylase. In contrast to the level of Asc in *vtc2*, Asc content in *vtc5* is 80% of the wild-type level [28]. Growth of *vtc2*/*vtc5* double mutant seedlings, however, is only possible when supplemented with Asc or l-galactose [28]. The cotyledons of *vtc2*/*vtc5* seedlings germinated in the absence of Asc or l-galactose undergo expansion but bleach within two weeks [28]. *vtc2*/*vtc5* plants maintained on Asc will grow to flowering, but the plants will bleach within one week in the absence of supplementation [28], suggesting that they experience an extreme level of photoinhibition in the absence of Asc. These results indicate the essential nature of Asc to prevent photooxidative damage. The absence of Asc would impair the deepoxidation of xanthophyll pigments catalyzed by VDE and its production of zeaxanthin required for the energy-dependent dissipation of excess absorbed light energy that composes the qE component of NPQ. The *npq1* mutant, which lacks VDE and therefore is unable to synthesize zeaxanthin in response to light, is also deficient in the qE component of NPQ but exhibits only a moderate increase in photoinhibition [103, 105]. Moreover, combining the *vtc2* mutation with either the *npq1 *or *npq4*, which lacks PsbS required for the qE component of NPQ, was only slightly more photosensitive than the *vtc2* mutant itself [103, 105]. These observations suggest that the role of Asc as a scavenger of ROS generated during photosynthesis is more important than its role in the xanthophyll cycle as a cofactor for VDE or the role of NPQ altogether.

### 4.3. Ascorbic Acid Regulates Abiotic and Biotic Stress Responses

As with conditions of excess light, environmental stress can also result in the production of ROS, often by limiting photosynthetic capacity leading to the overreduction of the photosystems and transfer of electrons from the photosynthetic machinery to molecular oxygen. Because of the critical role that Asc plays in detoxifying ROS generated during photosynthetic activity under normal growth conditions, it is not surprising that Asc is also important in determining the level of tolerance to many environmental stresses, including chilling, drought, salt, and exposure to heavy metals. The increased production of H_2_O_2_ under stress conditions, such as exposure to salt or water deficit, is a major contributor to the damage experienced by plants [84]. As H_2_O_2_ passes readily through cell membranes, it can cause damage at locations far from its site of generation [106].

A reduction in Asc content can increase sensitivity to salt stress. For example, the *vtc1* mutant has reduced tolerance to 200 mM NaCl as determined by CO_2_ assimilatory capacity and PSII function [107]. Under salt stress, *vtc1* plants had higher levels of H_2_O_2_ relative to wild-type plants despite having an elevated glutathione pool [107]. Although the transcript levels of MDAR and DHAR are induced by oxidative stress [108, 109], a reduction in MDAR and DHAR enzyme activities was observed in salt-stressed Arabidopsis [107], suggesting either that MDAR and DHAR protein levels are reduced in response to salt exposure, or their activities are inhibited by salt.

In an analysis of four interspecific *Prunus* hybrids subjected to water deficit, an increase in H_2_O_2_-related oxidative stress that occurred with the progressive loss of water from leaves was accompanied by an increase in the ascorbate-glutathione cycle-associated enzymes and their respective antioxidant substrates and was reversed following rewatering [110]. The Asc content in pumpkin (*Cucurbita pepo* L.) roots increased following exposure to 50 *μ*M aluminum sulfate, which correlated with an increase in the level of H_2_O_2_, APX activity, and ascorbate free radical reductase (AFRR) activity, whereas DHAR and glutathione reductase activity did not change [111].

A reduction in Asc content can also affect resistance to pathogens. The *vtc1* and *vtc2* mutants are more resistant to infection by *Pseudomonas syringae* and *Peronospora parasitica* as growth of the bacterial or fungal pathogen was substantially reduced [112]. The greater resistance correlated with a greater induction of the pathogenesis-related proteins PR-1 and PR-5 upon infection as well elevated salicylic acid levels [112], suggesting a faster induction of defense responses when Asc levels are low.

### 4.4. Ascorbic Acid Detoxifies Developmentally Generated ROS

ROS such as H_2_O_2_ are not only generated as a byproduct of photosynthesis following exposure to high light or stress conditions, but can be produced in large amounts during specific developmental stages. For example, H_2_O_2_ is generated in substantial quantities within the peroxisome of oilseeds as a byproduct of fatty acid *β*-oxidation during lipid catabolism that accompanies seedling growth [113, 114]. In order to detoxify H_2_O_2_, plant peroxisomes employ catalase in the matrix and a membrane-bound APX and MDAR, which detoxify the H_2_O_2_ through its Asc-dependent reduction [114–117]. The membrane association of the APX/MDAR system may serve to protect the peroxisomal membrane and reduce leakage of H_2_O_2_ into the cytosol [114, 116, 117]. Although apparently not required for growth under normal conditions [118], increasing expression from APX3, which is targeted to peroxisomes in Arabidopsis, increases tolerance against oxidative stress [119]. APX catalyzes the transfer of electrons from two molecules of Asc to H_2_O_2_ to form water and two molecules of MDHA. Thus, the enhanced ability to tolerate oxidative stress through an increase in the expression of the peroxisomal-targeted APX suggests that APX3 contributes significantly to detoxifying H_2_O_2_ before it can damage cellular components. That APX, and the Asc used by this enzyme, is important for responding to environmentally imposed oxidative stress was shown by the enhanced tolerance of tobacco overexpressing chloroplast-targeted APX to salt exposure or water stress [120].

The importance of peroxisomal APX activity in limiting H_2_O_2_-mediated cellular damage was further supported by the finding that peroxisomal-targeted MDAR also is critical in reducing cellular damage caused by H_2_O_2_ generated in the peroxisome. The Arabidopsis *sugar-dependent 2* (*sdp2*) mutant is deficient in MDAR4, a peroxisomal membrane isoform of MDAR, and is conditionally seedling-lethal as the seedlings are unable to catabolize storage oil [121]. *sdp2* mutants are impaired in fatty acid breakdown and exhibit increased levels of lipid peroxidation and protein oxidation. The *SDP1*-encoded triacylglycerol (TAG) lipase, which is responsible for a significant amount of the TAG lipase activity associated with oil body membranes [122], was inactivated through oxidative damage in *sdp2* mutant seedlings [121]. These findings suggest, that in the absence of MDAR4, some of the H_2_O_2_ generated in the peroxisome as a consequence of fatty acid *β*-oxidation during seed germination escapes and causes oxidative damage to oil bodies, including inactivating the SDP1 TAG lipase. The observation that peroxisomes and oil bodies cluster together, at least in *sdp2* seedlings, suggests that oil bodies are likely to be in close proximity to any H_2_O_2_ that may leak from the peroxisome. In contrast to oil bodies, peroxisomes appear less dependent on the APX/MDAR system for protection against H_2_O_2_, perhaps because catalase remains active within the peroxisomal matrix [121]. Therefore, the peroxisome-membrane associated MDAR isoform functions to reduce H_2_O_2_ leakage from peroxisome in order to protect TAG lipase activity and storage oil hydrolysis in the closely associated oil bodies during seedling growth.

### 4.5. Ascorbic Acid Regulates the Cell Cycle

In addition to its role as an antioxidant that reduces ROS, Asc also plays a role in regulating the cell cycle. An increase in the Asc pool size promotes cell division as did MDHA [17, 33, 123–127] whereas repression of l-galactono-1,4-lactone dehydrogenase (GalLDH) expression in tobacco BY-2 cell lines resulted in 30% less Asc and a reduced rate of cell division and growth [128]. Ascorbate oxidase mRNA and DHA decrease in the G1 phase of synchronous BY-2 cells [129]. During cell elongation, however, the level of ascorbate oxidase mRNA and activity increase as does the level of Asc and DHA, suggesting that the oxidation of Asc may be important during cell elongation [129].

Supporting the role of Asc in regulating the cell cycle is the inhibitory effect that an increase in the level of DHA has on cell-cycle progression but only if it is increased during the G_1_ and not during G_2_ phase [130, 131]. Asc promotes cell division by inducing G_1_ to S progression of cells within the quiescent center of onion roots [123, 126, 132–134]. Exogenous Asc also reversed the inhibition of cell division caused by lycorine treatment which reduces Asc content [132]. The addition of DHA reduced the mitotic activity of onion root meristems [124]. Interestingly, uptake of DHA in tobacco bright yellow-2 cell culture cells is highest during M phase and the M/G_1_ transition [135]. The effect of DHA appears, in part, to be due to its rapid reduction to Asc and the depletion of GSH, as the latter is a cofactor in the DHAR-mediated reduction of DHA. Supporting this conclusion is the observation that depletion of GSH through inhibiting its biosynthesis also inhibits cell-cycle progression [131]. Depletion of other possible reductants of DHA (e.g., thioredoxin), however, has also been proposed [130, 136]. This was suggested by the observations that increasing GSH did not prevent the inhibition of cell division by DHA and that a reduction in the level of GSH in combination with an increase in the level of DHA has an additive effect on inhibiting the cell cycle [130], indicating that their effects are independent. Given the effects of Asc, DHA, and GSH on the cell cycle, an oxidative stress checkpoint pathway has been proposed that controls cell-cycle progression by responding to one or more redox-sensing systems [137].

Exogenous DHA increased Asc content in *Lupinus albus* L. and *Allium cepa* L. root tips and inhibited cell proliferation, perhaps because of the transient depletion of GSH and oxidation of thiol-containing proteins [138]. Exogenous l-galactono-1,4-lactone, the precursor to Asc, also increased Asc content but did not cause the oxidation of thiol-containing proteins. Increasing Asc content in this manner stimulated growth [138]. These results suggest that DHA may inhibit cell division in roots through changes in the cellular redox state whereas Asc promotes root cell proliferation.

The quiescent center (QC) of the root is composed of a group of cells at the most distal part of the root proper just behind the root cap. It is this part of the root proper that represents the terminus for polar transport of auxin from the shoot. Auxin levels in the QC of *Zea mays *roots are higher than in adjacent meristematic cells while Asc content in the QC is substantially lower and ascorbate oxidase mRNA and activity higher relative to the adjacent meristematic cells [17]. Similarly, GSH content in the QC of Arabidopsis roots is lower than in surrounding tissues [139]. As ascorbate oxidase mRNA and activity were induced by exogenous auxin, these results suggest that the elevated levels of auxin in quiescent cells induce ascorbate oxidase expression which in turn reduces Asc content, thereby maintaining quiescent cells in their characteristic G_1_ state [17]. Exogenous Asc promoted a more rapid G_0_-G_1_ transition in embryo roots of *Pisum sativum L*. cv. Lincoln during germination but failed to promote cell division within cells of the QC [133]. In *Allium cepa* roots, however, Asc stimulated the mitotic activity of cells within the QC, as measured by the DNA synthesis activity, as well as stimulated cell proliferation in the root meristem and pericycle [123, 126, 127]. This suggests that Asc is necessary to promote cell-cycle progression for cells competent to pass through the G_1_/S phase checkpoint but may be insufficient to promote cell-cycle progression for cells that are not competent to pass this checkpoint, at least in some species.

How Asc functions to promote cell-cycle progression remains unknown. Asc is a cosubstrate of peptidyl-prolyl-4 hydroxylase, which catalyzes the hydroxylation of proline residues of cell wall-associated hydroxyproline-rich glycoproteins (HRGPs), for example, extensins and arabinogalactan proteins, which are involved in cell wall stiffening, signaling, and cell proliferation. The inhibition of peptidyl-prolyl hydroxylase with 3, 4-dl-dehydroproline reduced the hydroxyproline content of HRGP, altered cell growth, and inhibited cell-cycle progression in onion roots [140]. The low level of Asc in the QC may limit hydroxylation of proline residues and therefore the generation of HRGP peptidyl-prolyl hydroxylase which may contribute to the characteristic arrest of cell-cycle progression of cells in the QC.

### 4.6. Ascorbic Acid Regulates Cell Division during Embryo Development

The effect that Asc has on cell division is perhaps best illustrated by its effect on the first zygotic cell division during early plant embryo development. Embryo development normally initiates following the transverse division of a zygote into an apical, proembryo cell and a basal cell that gives rise to the suspensor, to generate one embryo per seed. Increasing the endogenous Asc content in tobacco through increasing expression of DHAR, however, induced monozygotic twinning and polycotyly [141]. Twinning induced by DHAR resulted from altered cell polarity and longitudinal instead of transverse cell division of the zygote, generating embryos of equal size. The direct injection of Asc into ovaries of wild-type tobacco phenocopied the DHAR-induced twinning and confirmed that it was the increase in Asc content resulting from the increased DHAR activity that was responsible for the twinning [141]. The effect of Asc on monozygotic twinning is developmentally limited to the first two days after pollination, consistent with its role in altering zygotic division. Similarly, polycotyly was induced when Asc levels were elevated just prior to cotyledon initiation [141]. The ability of Asc to promote monozygotic twinning and polycotyly can be understood by its effect on cell polarity and cell division. The promotion of zygotic division in a way that deviates from the normal transverse division results in the loss of the positional cues needed for the subsequent differentiation of the apical cell into the embryo and the basal cell into the suspensor. Consequently, the result is the generation of two genetically identical zygotes, each of which develops into an independent embryo. One of the twin zygotes can also divide again into two genetically identical zygotes, resulting in triplets [141]. Similarly, the alteration of cell division during the specification of cotyledon-forming fields during embryo development can increase the frequency of polycotyly. Although it is likely that Asc affects the division of other cells in a similar manner, it is perhaps only at critical stages of development, such as the first division of the zygote or during the specification of cotyledon-forming fields, that the control of cell division by Asc becomes readily apparent.

Perhaps related to its effects on cell division and elongation, Asc content is also correlated with growth. During seed development, the Asc pool size and Asc redox state (i.e., the ratio of Asc to DHA) change dramatically from a high level of Asc largely in its reduced state during early embryo development, followed by a decrease in the Asc redox state during cell elongation such that the level of DHA exceeds Asc, and finally the complete oxidation of Asc at seed maturity [142–144]. The DHA is once again rapidly reduced during germination to generate Asc and which is eventually augmented by an increase in Asc biosynthetic activity [143, 144]. The synthesis of Asc continues during leaf growth and declines with the decrease in leaf function as part of the aging process [145–147]. The *vtc1* mutant, with just 30% of the wild-type level of Asc, exhibits a significant reduction in growth [102]. A similar reduction in growth was observed in tobacco in which DHAR expression was repressed resulting in a lower ASC pool size and decrease in the redox state of Asc [146].

### 4.7. Ascorbic Acid Regulates Flowering Time

Analysis of *vtc* mutants has also suggested that Asc levels may affect flowering. Although the *vtc1* mutant was reported to exhibit a late flowering phenotype [102, 148], this was subsequently shown to be specific to growth under short-day length conditions [149]. Under long-day conditions, the *vtc1* mutant, as well as other *vtc* mutants, including *vtc2-1* and *vtc4-1*, exhibited an early flowering phenotype [150, 151]. Increasing Asc levels by feeding with l-galactono-1,4-lactone, the precursor to Asc, delayed flowering by 5 days [152]. The examination of transcripts associated with controlling flowering time revealed that circadian clock and photoperiod pathway genes are elevated in *vtc* mutants, suggesting that they are epistatic to these *vtc* mutants [151]. Changes in transcript levels for 171 genes were observed for the *vtc1* mutant, including many defense genes such as pathogenesis-related genes [148]. Abscisic acid (ABA) content was significantly increased in the *vtc1* mutant, up to 60% above WT levels, suggesting that some of the observed gene expression changes may be a result of this change in hormone balance [148].

## 5. Increasing l-Ascorbic Acid Content through Increased Biosynthesis

The most obvious approach to increasing Asc content in plants is to increase its biosynthesis. Depending on the biosynthetic enzyme employed, this approach has met with mixed results, possibly related to whether the catalyzed reaction represents a rate-limiting step. Overexpression of l-galactose dehydrogenase, the enzyme that converts l-galactose to l-galactono-1,4-lactone (Figure 1), in tobacco resulted in a 3.5-fold increase in l-galactose dehydrogenase activity but did not increase foliar Asc content although the suppression of l-galactose dehydrogenase expression in Arabidopsis resulted in reduced enzyme activity and foliar Asc content (Table 1) [37]. This observation suggests that the wild-type level of foliar l-galactose dehydrogenase activity is not rate limiting but may become so if its expression level is decreased, at least in tobacco. Whether this is true for other species will require a more comprehensive cross-species examination. In contrast, transient overexpression of an *Actinidia chinensis* (i.e., kiwifruit) GDP-l-galactose phosphorylase (within the d-mannose/l-galactose pathway) using agroinfection in tobacco leaves resulted in a 50-fold increase in GDP-l-galactose-d-mannose-1-phosphate guanyltransferase activity and more than a 3-fold increase in foliar Asc content [29] whereas its overexpression in stably-transformed Arabidopsis resulted in up to a 4-fold increase in Asc content [153]. Transient overexpression of kiwifruit GDP-l-galactose phosphorylase and GDP-mannose-3′, 5′-epimerase in agroinfected tobacco leaves resulted in up to a 7-fold increase in Asc content [153], suggesting that endogenous expression levels of these gene products may be rate limiting in leaves, at least in tobacco and possibly Arabidopsis.

Overexpression of d-galacturonic acid reductase (GalUR in the d-galacturonate pathway) from strawberry in Arabidopsis resulted in 2- to 3-fold increase in foliar Asc content (Table 1) [35]. The observation that GalUR can catalyze the production of l-ascorbic acid via d-galactonic acid and d-galacturonic acid does not indicate the extent to which this pathway contributes to Asc production in strawberry fruits or in other organs such as leaves. Radiotracer data in strawberry fruits had suggested that GalUR may be no more than a minor contributor to Asc biosynthesis [36]. However, a reduction in the expression of pectate lyase, which releases d-galacturonic acid during pectin solubilization in ripening strawberry fruits, reduced Asc levels suggesting that the d-galacturonate pathway does contribute to the Asc pool size in this fruit. Moreover, the demonstration that foliar Asc increases following overexpression of GalUR [35] suggests that the substrates for this pathway are present in leaves and therefore may provide another avenue to increase Asc content. The potential for this overexpression strategy will likely depend on the availability of d-galacturonic acid in a tissue and may therefore limit the usefulness of this approach to those organs where d-galacturonic acid is being released from pectin.

Transgenic tobacco and lettuce plants expressing a rat cDNA encoding l-gulono-1,4-lactone oxidase (GulLO) accumulated up to seven times more Asc than control plants [38]. Whether l-galactono-1,4-lactone or l-gulono-1,4-lactone served as the substrate for the rat GulLO is unknown. These results suggest, therefore, that either endogenous l-galactono-1,4-lactone served as the substrate for the rat GulLO or that l-gulono-1,4-lactone is present in plants. The observation that l-gulono-1,4-lactone can be converted into Asc in several plant species [34, 154, 155] supports the presence of this Asc biosynthetic pathway in plants. 

## 6. Regulation of l-Ascorbic Acid Recycling

### 6.1. Regulation of DHAR Expression

DHA is produced following the spontaneous disproportionation of MDHA to Asc and DHA. This disproportionation reaction can occur rapidly when the pH is low, for example, in the thylakoid lumen following exposure to light. As MDAR is absent from the thylakoid lumen and the ferredoxin of the photosynthetic electron transport chain is located on the stromal side of the thylakoid membrane and therefore unable to reduce MDHA generated in the lumen, all MDHA in the thylakoid lumen likely disproportionates into Asc and DHA. If DHA is not rapidly recycled to Asc; however, it undergoes irreversible hydrolysis to 2,3-diketogulonic acid which cannot be converted to Asc and therefore represents a loss to the Asc pool.

**Figure 2:**
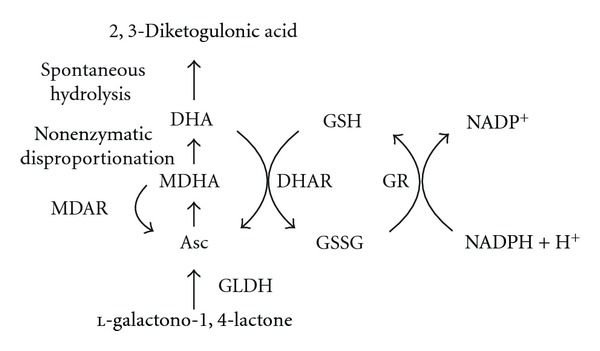
Role of DHAR and MDAR in l -ascorbic acid recycling. Asc is synthesized from l-galactono-1,4-lactone by l-galactono-1,4-lactone dehydrogenase (GLDH). When Asc is oxidized to monodehydroascorbate (MDHA), it can be reduced to Asc by monodehydroascorbate reductase (MDAR) or it can disproportionate non-enzymatically to Asc and dehydroascorbate (DHA). DHA spontaneously hydrolyzes to 2,3-diketogulonic acid unless salvaged by dehydroascorbate reductase (DHAR) which uses glutathione (GSH) as the reductant. Oxidized glutathione (GSSG) is reduced by glutathione reductase (GR) using NADPH as the reductant.

DHA is reduced to Asc by DHAR in a reaction requiring glutathione as the reductant [156, 157] (Figure 2). Because the apoplast contains little DHAR, DHA, which predominates in the apoplast, must reenter the cell for reduction. Reduction by DHAR allows the plant to recycle DHA before its irreversible hydrolysis, thereby recapturing the Asc before it is lost. Because Asc is the major reductant in plants, DHAR contributes to the regulation of the redox state in a plant. The importance of DHAR in preventing photoinhibition is no better demonstrated than in *Ficus microcarpa*, a tropical fig. This species contains little to no DHAR activity and is highly photosensitive to high light, resulting in photobleaching [158]. Consequently, the capacity to efficiently recycle DHA into Asc can be critical under conditions in which Asc is being rapidly consumed, for example, during exposure to light levels that exceed the photosynthetic capacity of a plant.

Five DHAR or DHAR-like genes have been annotated in Arabidopsis genome [159]. Three gene members: (*AtDHAR1*; At5g16710), (*AtDHAR3*; At1g75270), and (*AtDHAR5*; At1g19570) encode polypeptides for which other species contain obvious orthologs. Of the remaining two members, At5g36270 (*AtDHAR2*) may be a pseudogene as microarray data indicate that it is not expressed [159] and At1g19550 (*AtDHAR4*) is substantially smaller than canonical DHAR proteins as a result of multiple regions of the polypeptide missing from this member. The gene products of the three canonical DHAR genes likely localize to the cytoplasm (*AtDHAR3*; At1g75270), mitochondria (*AtDHAR5*; At1g19570), or chloroplast (*AtDHAR1*; At5g16710) [160]. Analysis of microarray data of individual DHAR genes revealed a complex pattern of regulation in response to environmental stresses or loss of other antioxidant enzymes [159]. *AtDHAR1* and *AtDHAR3 *expression was induced following exposure to cold or in plants suppressed for *CAT2* expression [159]. Their expression response differs, however, in that expression from* AtDHAR1* was induced by heat while *AtDHAR3 *expression was repressed. Moreover, expression from* AtDHAR3 *was induced by high light while *AtDHAR1 *expression was repressed [159]. Expression from all three canonical DHAR genes was repressed following exposure to salt or in plants lacking *CSD2* expression. *CSD2* encodes a SOD, and suppression of its expression leads to high-light stress response even under low light [161]. These observations indicate that the regulation of individual *AtDHAR* gene family members has evolved to respond to specific abiotic cues.

*AtDHAR3* expression increased 32-fold at the mRNA level following exposure to 200 ppb ozone with no change in expression of the isoforms targeted to the mitochondria or chloroplast [160]. In the absence of *AtDHAR3* expression, plants lacked cytosolic DHAR activity and were more sensitive to ozone [160], consistent with the induction of this gene following ozone exposure. Although the total pool size of Asc and GSH was unchanged in the absence of *AtDHAR3* expression, the Asc redox state was decreased by more than 2-fold due to an increase in DHA. A 61.5% reduction in the level of apoplastic Asc was also observed in this DHAR mutant. As there is no DHAR activity in the apoplast and therefore DHA must be transported from the apoplast to the cytoplasm for recycling back into Asc, a reduction in the cytosolic isoform of DHAR would be expected to affect the Asc redox state of the apoplast. The effect that a reduction in DHAR expression has on reducing the apoplastic Asc content and on increasing ozone sensitivity is in good agreement with earlier observations made in tobacco [162].

In an analysis of the regulation of the ascorbate-glutathione cycle enzymes during germination of barley, DHAR, glutathione reductase, and MDAR activities were already present by 4 hr after imbibition. In the case of DHAR, its activity decreased until 72 hr after imbibition but increased again by 144 hr after imbibition where GR and MDAR activities increased modestly by 144 hr after imbibition [163]. No APX activity was detected in mature seeds, but it was detected 24 hr after imbibition and increased a further 14-fold up to 144 hr after imbibition. The lack of correlation between DHAR protein levels and the observed changes at the level of its activity suggested possible posttranslational regulation.

GalLDH, DHAR, APX, MDAR, and GR activities in potato leaves exhibited a transient increase (with an accompanying increase in the Asc pool size) following exposure to either high (40°C) or low (5°C) temperature which was then followed by a decrease in these activities and in Asc content but an increase in the level of foliar MDA and H_2_O_2_ [164]. Similarly, the level of transcript, protein, and activity of DHAR increased in rice seedlings exposed to elevated temperature (i.e., 40°C) [165]. A lower Asc redox state was observed in leaves of the drought-sensitive wheat genotype, Cappelle Desprez, than in the drought-tolerant Plainsman V genotype in response to mild water deficit [166]. These observations indicate that the expression of enzymes involved in ROS detoxification is induced as part of the early response to many abiotic stresses but is also repressed with prolonged stress conditions.

### 6.2. Regulation of MDAR Expression

The oxidation of Asc produces the short-lived radical MDHA (Figure 2). MDHA is generated by several means including as a product of the reaction catalyzed by APX; following the reaction of Asc with any O_2_^∙−^,  OH^∙^, or thiyl radical not reduced by the normal scavenging systems present in the chloroplast; following the reaction with organic radicals such as the tocopherol radical in order to regenerate tocopherol in the thylakoids [91]. Within the thylakoid lumen, MDHA is also produced by VDE or following the donation of electrons from Asc to PSI or PSII [86, 167]. MDHA can be recycled to Asc by ferredoxin (Fd) in the chloroplast stroma or by MDAR in the chloroplast stroma or cytosol [91]. Within the thylakoid lumen, MDHA cannot be reduced by Fd or MDAR as neither is present in the luminal space. Instead, MDHA spontaneously disproportionates rapidly to Asc and DHA when the pH of the lumen is low, that is, during exposure to light which results in the transport of protons from the stroma across the thylakoid membrane into the luminal space [85, 91]. In the stroma where the pH is higher, particularly following exposure to light, MDHA disproportionates much more slowly. In this case, Fd or MDAR is available to reduce MDHA to Asc. PsaC of the PSI complex is responsible for reducing Fd, which, in the form of photoreduced Fd, donates electrons to NADP^+^ in a reaction catalyzed by Fd-NADP^+^ reductase (FNR). Photoreduced Fd (redFd) can donate electrons to MDHA to reduce it to Asc at a rate of 10^7^ M^−1^ s^−1^ [168]. Although redFd reduces both NADP^+^ and MDHA, the rate of MDHA reduction by redFd is 34-fold greater than NADP^+^ [168]. MDHA can also be reduced by MDAR, a flavin adenine dinucleotide (FAD) enzyme, which uses NADH (*K*_*m*_; 5 *μ*M) or NADPH (*K*_*m*_; 22–200 *μ*M) as the source of the electrons [169]. Because redFd preferentially reduces MDHA over NADP^+^, the bulk of MDHA reduction by the thylakoidal scavenging system likely occurs through Fd. In the stroma, however, MDAR is expected to contribute significantly to the reduction of MDHA as part of the stromal scavenging system.

The Arabidopsis genome contains five genes that encode MDAR enzymes [159]. Analysis of microarray data for the individual MDAR gene family members revealed a complex pattern of regulation in response to environmental stresses or loss of other antioxidant enzymes [159]. However, expression of all MDAR genes was repressed following exposure to heat [159]. Expression of *AtMDAR3*, *AtMDAR4*, and *AtMDAR5* was similarly induced by water deficit or exposure to high light [159]. These same genes were also induced in the absence of *APX1* expression [159]. Loss of *APX1* expression resulted in a lack of stomatal opening in response to light, as well as lower photosynthetic activity and enhanced induction of heat shock proteins following exposure to moderate light stress [170]. *AtMDAR4* was induced by most stresses including water deficit, cold, salt, and high light as well in plants lacking *APX1* expression or suppressed for *CAT2* expression. In other species, a cytosolic MDAR was induced in tomato following wounding [109] while MDAR activity increased in *Pinus sylvestris* roots following exposure to cadmium but declined in roots of poplar hybrids (*Populus* x *Canescens*) exposed to this heavy metal [171, 172]. MDAR activity increased in rice seedlings subjected to water deficit [173].

MDAR is expressed solely from nuclear genes, but these encode isoforms that are targeted to the cytosol, chloroplast, mitochondria, and peroxisomes. Those MDAR isozymes that are targeted to peroxisomes, chloroplasts, and mitochondria accompany APX and function as a scavenging system to detoxify any H_2_O_2_ that has not undergone disproportionation normally catalyzed by peroxisomal catalase [174]. Dual targeting of MDAR to chloroplasts and mitochondria was observed in Arabidopsis and resulted from the use of multiple transcription start sites, producing a seven amino acid N-terminal extension in the mitochondrial-targeted form of the protein [175]. The 47-kDa AtMDAR1 and 54-kDa AtMDAR4 isoforms contain a C-terminal sequence that is responsible for matrix (PTS1) and membrane peroxisomal targeting, respectively [176]. Expression from MDAR1, a peroxisomal-targeted MDAR in pea, was upregulated most following exposure to cold and to a lower extent by wounding or treatment with the herbicide 2,4-dichlorophenoxyacetic acid [177].

## 7. Increasing l-Ascorbic Acid Content through Improved Recycling

### 7.1. Increasing DHAR Expression

DHAR is expressed in rate-limiting amounts and contributes significantly to establishing the cellular Asc redox state [11, 146, 162, 178]. The first attempt to increase the Asc content in plants through the overexpression of DHAR employed the expression of a human DHAR gene in tobacco chloroplasts [179]. The transgenic plants had more than a 2-fold increase in DHAR activity and a 1.43-fold increase in GR activity (Table 2). Although the plants exhibited a 2-fold increase in the Asc redox state, the Asc content was not significantly changed [180]. Moreover, the GSH redox state (i.e., the ratio of GSH to GSSG) of the plants was substantially lower due to a decrease in GSH and an increase in GSSG [179].

The first demonstration that increasing DHAR expression could elevate Asc content was shown in transgenic tobacco and maize expressing a cytosolic wheat DHAR [11]. Following the introduction of the wheat DHAR cDNA into tobacco and maize, DHAR activity increased 11–100 fold and was accompanied by increases in Asc and its redox state (Table 2), consistent with the function of DHAR in reducing DHA to Asc. In expanding tobacco leaves, the overexpression of DHAR resulted in a simultaneous increase in Asc and a decrease in DHA. Similar increases in the Asc pool size and redox state were observed in maize leaves and developing kernels [11], demonstrating that changes in Asc can be made in photosynthetic and nonphotosynthetic organs. When the level of Asc and DHA was measured in the apoplast of DHAR-overexpressing tobacco leaves, increases in Asc content and its redox state were observed [178], demonstrating that the level of cytosolic DHAR serves to regulate the symplastic and apoplastic Asc pool size and redox state. The increase in DHAR expression did not appear to affect Asc biosynthesis as no increase in l-galactono-1,4-lactone dehydrogenase activity was observed. An increase in the pool size and redox state of GSH, however, did occur [11]. As the cellular concentration of Asc is determined by the rate of its synthesis and decay, the observed increase in Asc can be understood through the recycling function of DHAR which recycles DHA to Asc before it is lost through decay. Thus, the increase in Asc content and the reduction in DHA content in DHAR overexpressing plants alter not only the Asc pool size but also its redox state. The ability to improve the efficiency of Asc recycling through increases in DHAR expression indicates that the level of DHAR expression in these species is rate limiting. Therefore, the likelihood of successfully increasing the Asc content of a plant by this means will depend on whether the level of DHAR expression in a species is rate limiting.

Increasing DHAR expression as a viable strategy to increase Asc levels and/or the redox state of Asc in plants has been validated in a number of studies. Approaches have included increasing DHAR expression in the cytosol or in the chloroplast, as DHAR isoforms are present in both with the chloroplast isoform a product of a nuclear gene. Transgenic tobacco expressing a cytosolic DHAR from Arabidopsis had a 2.3- to 3.1-fold increase in DHAR activity and a 1.9- to 2.1-fold increase in Asc with a 2.4–2.6-fold increase in the Asc redox state [182]. Expression of an Arabidopsis cytosolic DHAR in Arabidopsis increased DHAR expression by 1.5- to 5.4-fold and was accompanied by an increase in foliar Asc by 2 to 4.25-fold [187]. The Asc redox state also increased 3- to 16-fold relative to the wild type. Tobacco overexpressing an Arabidopsis cytosolic DHAR exhibited an increase in Asc and its redox state with no change to the pool size or redox state of GSH [183]. A slight increase in DHAR expression following introduction of a rice cytosolic DHAR cDNA into Arabidopsis resulted in a slight increase in Asc content [188]. Expression of a rice DHAR in transformed tobacco chloroplasts increased foliar Asc levels slightly which was further increased in double chloroplast transgenics expressing GR and DHAR [184]. The Asc redox state increased more substantially due to a simultaneous increase in Asc and a decrease in DHA.

In an effort to bolster the nutritional value of maize endosperm by increasing synthesis of three vitamins, transgenic corn was generated expressing a maize phytoene synthase (*psy1*) under the control of a wheat glutenin promoter as well as a *Pantoea ananatis* carotene desaturase (*crtI*), a rice DHAR, and an *E*. *coli* GTP cyclohydrolase (*folE*), each under the control of a barley d-hordein promoter [13]. Together, the *psy1* and *crtI* genes function to increase *β*-carotene levels, while DHAR increases Asc content, and the *folE* gene functions to increase folate levels. These transgenes resulted in a 169-fold increase in *β*-carotene, a 6-fold increase in Asc, and doubling of folate content [13].

A sesame DHAR cDNA was introduced into potato under the control of either the constitutively active CaMV 35S promoter or the patatin promoter, which directs expression specifically in tubers [185]. The patatin promoter directs high expression of the sesame DHAR in tubers but not in leaves whereas the CaMV 35S promoter resulted in expression in leaves to a higher level than in tubers. Asc content in Patatin::DHAR tubers increased from 1.1- to 1.3-fold with no increase in leaves whereas Asc content in CaMV35S::DHAR leaves increased 1.5-fold while Asc content in CaMV35S::DHAR tubers increased 1.6-fold [185]. In a second study using potato, expression of a potato cytosolic DHAR from the CaMV 35S promoter increased the Asc content in leaves by more than 1.6-fold and in tubers by more than 1.2-fold [186]. This correlates with the expression profile of this cytosolic DHAR where it is expressed in leaves, stems, and tubers, but its expression is higher in tubers and lower in leaves and declines with leaf age [186]. In contrast, expression of a chloroplast-localized DHAR from potato increased DHAR activity and Asc content in leaves by up to 1.5-fold but not in tubers correlating with its expression profile in which it is normally expressed highest in leaves up to their maturity but is not expressed in tubers [186]. These results indicate that increasing DHAR expression in chloroplasts as a means to increase Asc may be limited to photosynthetically active tissues whereas increasing cytosolic DHAR expression may provide an approach for increasing Asc content in a wider range of organs.

### 7.2. Increasing MDAR Expression

Expression of a cytosolic tomato DHAR from a constitutive promoter in tomato (var. Micro-Tom) resulted in a 1.6-fold increase in Asc in mature green and red ripe fruit from plants grown under low light, but foliar Asc was unchanged [189]. A similar approach to overexpress a cytosolic tomato MDAR resulted in a significantly reduced Asc content in mature green tomato fruits but an unaltered Asc content in leaves (Table 3) [189]. However, a correlation between MDAR expression in tomato and improved chilling tolerance was observed in fruit [190], and increasing MDAR expression in tobacco improved tolerance against salt and osmotic stresses [191]. Expression of a tomato chloroplast-targeted MDAR in tomato increased MDAR activity by about 1.9-fold and increased foliar Asc by 1.2-fold with a corresponding decrease in DHA, resulting in an approximate doubling of the Asc redox state [192]. Tobacco overexpressing an Arabidopsiscytosolic MDAR had a slightly higher Asc content and redox state with no change to the GSH pool size or redox state [183]. Compared to work on DHAR, there are relatively fewer reports on the effect that increasing MDAR expression has on Asc content. The reports to date do suggest, however, that targeting MDAR expression may increase Asc content but that it may not be as successful as targeting DHAR expression.

## 8. Consequences of Altering DHAR Expression in Plants

### 8.1. DHAR Expression May Affect the Level of Antioxidants other than Ascorbic Acid

Altering Asc content and/or its redox state might be expected to impact the level of other antioxidants or the activities of those enzymes that generate them. To date, although most studies have observed that increasing DHAR expression results in an increase in Asc content and/or in Asc redox state, there is less agreement on its possible secondary effects on other antioxidants. Increases in GSH content and its redox state were observed in DHAR-overexpressing tobacco and maize with no significant change in the activities of GR, APX, CAT, and SOD [11]. Similar effects on GSH content and its redox state were observed in Arabidopsis expressing an Arabidopsis cytosolic DHAR [187]. The increase in GSH that accompanies an increase in Asc suggests a coordinate balance between these two antioxidants, which is not unexpected given that GSH is required by DHAR. Because glutathione is present at a concentration that is one to two orders of magnitude lower than that of Asc [79], an increase in the level of Asc may require a corresponding increase in GSH, suggesting that changes in Asc may act as a signal for changes in the GSH pool size. Expression of human DHAR gene in tobacco chloroplasts, however, resulted in a 1.43-fold increase in GR activity and a substantial decrease in the GSH redox state due to a lower level of GSH [179]. Additional work is needed to fully characterize the effect that an increase in the expression of cytosolic versus chloroplastic DHAR has on other antioxidants and those enzymes that generate them.

### 8.2. DHAR Expression Regulates Tolerance to Environmental ROS

Because Asc is the most abundant antioxidant in plants, most studies examining the consequences of altering DHAR expression have focused on alterations in the response to environmental stress known to generate ROS, for example, high light, ozone, chilling, salt, or drought. The first convincing demonstration that Asc plays a critical role in protecting a plant from environmental ROS was shown with the *vtc *mutants of Arabidopsis [27, 101]. The *vtc1* mutant, with just 25%–30% of the wild-type level of Asc, is hypersensitive to ozone and sulfur dioxide [25, 27, 102]. As *vtc1* plants contain a higher oxidative load relative to wild-type plants when exposed to stress conditions such as salt even though they contain more GSH [107], their lower Asc content is clearly responsible for the impairment in detoxifying stress-related ROS.

That the endogenous level of apoplastic Asc is important in detoxifying ozone was shown in tobacco in which the level of apoplastic Asc was specifically altered [193]. A decrease in the Asc redox state following overexpressing an apoplastic-localized ascorbate oxidase (AO) from cucumber in transgenic tobacco increased its ozone sensitivity [193]. The increase in AO expression did not affect the total amount of ascorbate (i.e., Asc and DHA) in the apoplast or symplast but did convert virtually all apoplastic Asc to DHA thus eliminating the potential for detoxification of ozone in the apoplast. The increase in apoplastic DHA also resulted in a lower symplastic Asc redox state which would further compromise the ability to detoxify the ozone invading the cytosol. The observation that the level of symplastic glutathione was not reduced, and its redox state was actually higher in tobacco overexpressing the apoplastic-localized AO [193] suggested that the increase in ozone sensitivity was likely due to a lower Asc redox state alone.

Plants can limit damage caused by environmental ROS, such as ozone, either by reducing its diffusion into the leaf interior (i.e., avoidance) or detoxification of any that does enter (i.e., tolerance) [194]. When ozone does enter the plant, it rapidly degrades into hydroxyl radicals and other ROS that can be converted to H_2_O_2_ [92, 195]. ROS are first observed in guard cell chloroplasts and membranes but spread to neighboring cells [196]. As ozone is converted to H_2_O_2_ in the apoplast or following its entry into the cytoplasm [92], an increase in the amount of H_2_O_2_ present in guard cells promotes stomatal closure thereby limiting further entry of ozone into the leaf interior. Avoidance strategies, therefore, focus on limiting ozone diffusion into a leaf. In contrast, tolerance strategies involve ozone detoxification, either through chemical reaction with apoplastic Asc or enzymatically, for example, by APX, following the conversion of ozone to H_2_O_2_ in the cytosol.

Increasing Asc recycling by increasing DHAR expression reduces the level of H_2_O_2_ in guard cells, resulting in a reduction in the responsiveness of guard cells to ozone [162, 178]. As a consequence, this slower responsiveness of guard cells allows more ozone to diffuse into the leaf interior [162, 178]. At the same time, however, the increase in DHAR activity in the other cells of the leaf increases the Asc content of their apoplast and symplast and thus increases their ability to detoxify the ozone which does enter the leaf interior [162, 178]. Conversely, a reduction in Asc recycling through the suppression of DHAR expression increases the responsiveness of guard cells to ozone thereby limiting ozone diffusion into the leaf interior [162, 178]. The decrease in DHAR expression, however, lowers the Asc content and redox state of leaf cells and thus reduces their ability to detoxify invading ozone.

The generation of plants in which DHAR expression was either increased or suppressed provided a means to address the question of whether altering guard cell responsiveness or the foliar Asc content itself is more useful in preventing the oxidative stress imposed by environmental ROS. Although guard cells of DHAR-overexpressing tobacco were less responsive to ozone and allowed greater diffusion of ozone, the greater Asc content of the leaves reduced their oxidative load (i.e., a lower level of foliar and apoplastic H_2_O_2_) such that they had a lower induction of ROS-related enzyme activities, more chlorophyll, and a higher level of photosynthetic activity following an acute exposure to ozone than control plants [162]. Conversely, guard cells of DHAR-suppressed tobacco were hyperresponsive to ozone which limited its diffusion into the leaf interior but also reduced photosynthetic activity of the leaf [162]. Therefore, increasing DHAR expression allowed more ozone to enter the leaf interior but also provided the additional capacity to reduce the invading ozone. This strategy provided greater protection (i.e., greater tolerance) against oxidative damage imposed by this environmental ROS without compromising photosynthetic activity than did reducing total stomatal area (i.e., greater avoidance) which limited photosynthetic activity in addition to ozone diffusion [162]. These results demonstrated that higher foliar Asc confers a greater degree of protection against environmental oxidative damage than does increasing guard cell responsiveness.

These observations were validated in transgenic tobacco expressing a cytosolic Arabidopsis DHAR that had a 2-fold increase in Asc content and exhibited enhanced tolerance to ozone (as well as drought, salt, or polyethylene glycol) as measured by photosynthetic activity [182]. Moreover, the greater sensitivity to ozone exhibited by the Arabidopsis *AtDHAR3* mutant, which fails to express cytosolic DHAR activity [160], serves as additional evidence indicating the importance of DHAR in responding to environmental ROS. The observation that the *AtDHAR3* mutant has a lower Asc redox state but not pool size suggests that it is the efficiency of Asc recycling that is critical to limiting oxidative damage. The induction of *AtDHAR3* expression following ozone exposure [160] is further indication of role the DHAR plays in this response program. These results are in general agreement with reports indicating the importance of Asc in providing resistance against oxidative stress imposed by ozone [197, 198].

### 8.3. DHAR Expression Regulates Tolerance to Other Abiotic Stresses

In addition to ozone, increasing DHAR expression confers greater tolerance to other environmental stresses. Tobacco expressing a human DHAR gene in chloroplasts increased the Asc redox state without altering the Asc pool size [180] and following exposure to 5 *μ*M methyl viologen or 200 mM H_2_O_2_, the plants exhibited a 40% and 25% reduction in membrane damage relative to the control, respectively. Transgenic seedlings also had enhanced tolerance to low temperature (15°C) and 100 mM NaCl [180]. Combining expression of a chloroplast-localized DHAR with the expression of a chloroplast-localized SOD (i.e., a CuZnSOD) and APX had a similar effect on increasing DHAR activity as well as the Asc and GSH redox states relative to plants expressing just the chloroplast-localized CuZnSOD and APX [199]. The combination of these three antioxidant enzymes resulted in enhanced tolerance to paraquat and 100 mM salt, indicating that the beneficial effect of increasing DHAR expression can be used in a combinatorial approach with other enzymes involved in oxidative stress, and its inclusion provides a significant improvement over approaches that employ just CuZnSOD and APX.

The simultaneous expression in tobacco of two pairs of chloroplast-localized enzymes, that is, an *E*. *coli* GR in combination with either an *E*. *coli* glutathione-S-transferase (GST) that exhibits GSH-dependent peroxidase activity or a rice DHAR, resulted in an increase in Asc and GSH content as well as their respective redox states [184]. Enhanced tolerance to salt and cold was observed for these transformants [184]. Leaf discs from plants overexpressing a combination of DHAR and GR or GST and GR were more efficient at reducing H_2_O_2_ during chilling stress than were leaf discs from plants overexpressing DHAR or GST alone. Although expression of each single transgene failed to confer tolerance to methyl viologen-induced oxidative stress, the combinatorial expression of DHAR and GR or GST and GR did [184], suggesting that increasing chloroplastic DHAR and GR recycling activities increases tolerance to abiotic stress. No difference in sensitivity to CdCl_2_ or ZnSO_4_ was observed in the transplastomic lines relative to the WT, and only a slight increase in salt tolerance was observed [184].

Tobacco overexpressing Arabidopsis cytosolic DHAR exhibited greater tolerance to aluminum, without affecting the distribution or accumulation of Al in root tips after 24 hr of exposure [183]. DHAR-overexpressing plants maintained greater Asc content in roots prior to and following Al treatment and maintained a higher level of APX activity following Al treatment than wild-type plants. Roots of DHAR-overexpressing plants also had lower levels of H_2_O_2_, lipid peroxidation, and DNA damage and had better root growth relative to wild-type plants [183]. Increasing APX expression or suppressing AO expression appears to have a similar effect on tobacco as overexpression of APX in tobacco chloroplasts enhanced tolerance to salt stress and water deficit [120] whereas suppression of AO expression resulted in a greater Asc redox state, higher photosynthetic activity, and a reduction in H_2_O_2_ [200].

In Arabidopsis, increasing DHAR expression increased Asc content and its redox state with an accompanying increase in GSH content and its redox state [187]. These DHAR-expressing plants retained more Asc and chlorophyll with less membrane damage than did control plants following exposure to high light and high temperature or following treatment with paraquat [187]. Overexpression of a rice DHAR in Arabidopsis also increased tolerance to salt stress during germination although the increase in DHAR activity and total ascorbate was small [188], suggesting that rice may be highly sensitive to changes in DHAR activity and total ascorbate. Growth beyond the seedling stage was not examined. No difference in cold tolerance was observed.

### 8.4. DHAR Expression Regulates Guard Cell Responsiveness

Although ROS can be highly damaging and altering Asc content through changes in DHAR expression can significantly impact the level of damage sustained upon exposure to ROS-generating environmental stresses, ROS also serve important signaling functions providing information about changes in the external environment. H_2_O_2_ has been implicated to serve a signaling role in guard cells which determine the extent of gas exchange and transpiration. Stomatal pores in many species open in the morning but close in the afternoon to limit water loss [201]. Stomatal pores also close in response to conditions of limited water which is mediated through the action of ABA. ABA signaling results in an increase in cytosolic Ca^2+^ concentration from H_2_O_2_-activated Ca^2+^ channels and from release from intracellular stores [94, 95, 202]. Water stress causes an increase in H_2_O_2_ production, which in turn serves as a signaling intermediate to promote stomatal closure [202]. Although tobacco overexpressing DHAR grew normally under well-watered conditions, increasing the Asc redox state resulted in an increase in transpiration rate and rate of water loss under normal and water stress conditions whereas lowering the Asc redox state by suppressing DHAR expression decreased water loss up to 30% [178]. In addition, following a severe water stress which caused leaf wilting in DHAR-overexpressing plants and in which the rate of CO_2_ assimilation was substantially reduced, DHAR-suppressed leaves retained turgor, and the rate of CO_2_ assimilation was only slightly reduced relative to well-watered conditions [178]. Therefore, increasing DHAR expression increases the Asc redox state of guard cells and results in a reduced responsiveness to water stress. This in turn results in an enhanced rate of water loss as a consequence of the increase in the total open stomatal area. Conversely, suppressing DHAR expression reduces transpiration and CO_2_ assimilation under normal growth conditions as a consequence of the reduction in the open stomatal area but also reduces water loss resulting in increased drought tolerance.

The role of Asc in guard cell functioning can thus be understood through its role as a scavenger of H_2_O_2_ whereby the balance between H_2_O_2_ production and the Asc redox state establishes whether the H_2_O_2_ concentration rises to a level that can trigger stomatal closure. A diurnal increase in H_2_O_2_ occurs such that the level of H_2_O_2_ rises during the course of the day and declines again during the night [178]. This diurnal change in the level of H_2_O_2_ is likely a result of photosynthetic-related processes such as photorespiration and oxygen photoreduction (e.g., the Mehler peroxidase reaction). These function to maintain a flow of electrons through PSI in order to prevent the overreduction of the photosystems and photodamage [91]. In the Mehler reaction, superoxide is generated from the transfer of electrons from PSI to oxygen which SOD disproportionates to O_2_ and H_2_O_2_. APX then reduces H_2_O_2_ to water using Asc as the reductant. ABA can also elicit H_2_O_2_ production as part of the signaling required to promote stomatal closure [202]. As DHAR is present in rate-limiting amounts, the observed diurnal increase in DHA that occurs during the day likely reflects the consumption of Asc during the reduction of H_2_O_2_ and the inability of the endogenous level of DHAR to efficiently reduce the DHA to Asc. Thus, under conditions of excess light, the consumption of Asc in the water-water cycle and the inability of a rate-limiting amount of DHAR to regenerate Asc efficiently allows the concentration of H_2_O_2_ to increase to a level that signals stomatal closure. Overexpression of DHAR provides a larger reservoir of Asc while also enabling more efficient regeneration of Asc so that H_2_O_2_ is maintained at a lower level and therefore delays stomatal closure. This explains the greater open stomatal area, increased stomatal conductance, higher transpiration rate, higher rate of water loss, decreased tolerance to water stress, and reduced guard cell responsiveness to ABA and H_2_O_2_ signaling that is observed in DHAR-overexpressing plants [178]. Conversely, reducing DHAR expression to below wild-type levels decreases the Asc redox state and reduces the efficiency of Asc regeneration when it is being consumed leading to an elevated accumulation of H_2_O_2_, which in turn triggers a greater degree of stomatal closure even under nonstress conditions [178]. The signaling function of H_2_O_2_ in guard cells, therefore, is controlled by the rate of its production and the rate of its removal in which the level of Asc and DHAR play critical roles.

### 8.5. DHAR Expression Regulates Plant Growth

In addition to being hypersensitive to ozone, sulfur dioxide, or UVB light, *vtc1* plants exhibit slower shoot growth, smaller leaves, and reduced shoot fresh weight and dry weight [102], suggesting that changes to the Asc pool size affect plant growth. Plants suppressed in DHAR expression exhibited a slower rate of leaf expansion, slower shoot growth, delayed flowering time, and reduced foliar dry weight [146]. These phenotypes correlated with reduced leaf function as measured by the preferential loss of chlorophyll a, a reduced level of the ribulose bisphosphate carboxylase/oxygenase large subunit (RbcL), and a lower rate of CO_2_ assimilation in young leaves and a premature onset of senescence in mature leaves [146]. Although reducing DHAR expression reduces stomatal conductance which might be expected to limit CO_2_ diffusion into the leaf interior, the reduced rate of CO_2_ assimilation was observed in expanding and newly expanded leaves in which stomatal conductance was not substantially affected [146]. Moreover, despite the lower stomatal conductance in older DHAR-suppressed leaves, the substomatal CO_2_ concentration was higher than in control leaves, suggesting that CO_2_ diffusion into the leaf interior was not being limited but rather CO_2_ was not being used efficiently. The reduced rate of expansion observed in young leaves in DHAR-suppressed plants is a likely a consequence of the accelerated loss of leaf function in the fully expanded leaves that may have prematurely reduced the photosynthate that could be made available to the sink tissues. This would also explain the slower growth rate of the plant as measured by plant height and leaf number. The effect of DHAR expression on leaf aging inversely correlated with the level of lipid peroxidation indicating that the efficiency of Asc recycling was important in regulating ROS-mediated damage [146]. A similar effect on plant growth was observed in tomato in which expression of the enzyme that catalyzes the last step in the principle Asc biosynthetic pathway was repressed using an antisense approach [203]. The inhibition of up to 80% of expression of l-galactono-1,4-lactone dehydrogenase (GalLDH) resulted in a lower Asc redox state without altering the total ascorbate content in tomato. Plant growth rate was decreased with reductions in the final size of leaves and fruits which was a consequence of reduced cell expansion [203]. Repression of GalLDH expression in tobacco BY-2 cell lines resulted in 30% less Asc, and the lines exhibited a reduction in cell division and growth rate [128].

In contrast, increasing DHAR expression resulted in higher levels of RbcL and chlorophyll and a higher rate of CO_2_ assimilation in presenescent leaves [146]. However, increasing DHAR expression did not substantially increase leaf expansion or overall plant growth, indicating that the endogenous level of DHAR expression is sufficient to provide the level of Asc recycling that is not rate limiting for the photosynthetic activity needed to support maximum growth. This is in contrast to the role of DHAR in regulating guard cell function whereby the overexpression of DHAR results in a wilty phenotype [178].

### 8.6. DHAR Expression Regulates Photosynthetic Activity

Reducing DHAR expression resulted in the alteration of xanthophyll pigments that could account for a reduced qE and an increased photoinhibition (qI) [181]. A reduction in the quantum yield of PSII (*ϕ*PSII) and the electron transport rate (ETR) accompanied the decrease in NPQ, while the level of ROS increased. Leaves with reduced DHAR expression exhibited poor recovery following exposure to high light, indicating that they experienced a greater degree of photoinhibition [181]. Increasing DHAR expression resulted in an increase in the xanthophyll pigment and chlorophyll pool size, as well as in the ETR and in the rate of CO_2_ assimilation, particularly at high-light intensities, while the level of ROS was reduced [181]. The increase in DHAR expression resulted in less photoinhibition following exposure to high light. Thus DHAR functions to ensure the appropriate level of induction of NPQ and contributes to photoprotection during leaf aging. These observations support the conclusion that through its Asc recycling function, DHAR acts to regulate the basal level of ROS present in leaves during their development and, as a consequence, regulates the rate of leaf aging as defined by its photosynthetic activity. Thus, these observations support the conclusion that an important function of DHAR in leaves is to maintain photosynthetic functioning by limiting ROS-mediated damage.

## 9. Consequences of Altering MDAR Expression in Plants

Relative to the work with DHAR or Asc biosynthetic mutants, fewer studies on how changes in MDAR expression affect plant growth and function have been reported. The observation that MDAR overexpression appears to affect Asc levels to smaller extent than does the overexpression of DHAR may account to some extent for these fewer reports. MDAR null mutants, however, have demonstrated the critical role that this activity can play. As mentioned previously, loss of expression of MDAR4, as in the *sdp2* mutant of Arabidopsis, is conditionally seedling-lethal as the seeds are unable to catabolize storage oil [121]. This peroxisome-membrane associated MDAR isoform functions to reduce H_2_O_2_ leakage from peroxisome in order to protect TAG lipase activity and storage oil hydrolysis in the closely associated oil bodies during seedling growth. Loss of its activity results in H_2_O_2_-mediated inactivation of TAG lipase activity and therefore the inability to use storage oil needed to support seedling growth [121].

Tomato seedlings overexpressing a chloroplast-targeted tomato MDAR, which had a slight increase in Asc content and an approximate doubling of the Asc redox state, had a lower oxidative load (as measured by H_2_O_2_), lower thiobarbituric acid reactive substance (TBARS) content (a measure of membrane damage), a higher net photosynthetic rate (*P*_*n*_), higher maximal photochemical efficiency of PSII (*F*_*v*_/*F*_*m*_)- and greater fresh weight when subjected to low- (4°C for 7 days) or high- (40°C for 7 days) temperature stress [192]. Similar results were obtained when the plants were treated with methyl viologen. In contrast, antisense transgenic lines exhibiting 54–60% of the wild-type level of MDAR activity with a 21–27% reduction in Asc and a 2-fold decrease in the Asc redox state had a higher oxidative load, higher TBARS content, lower *P*_*n*_, lower *F*_*v*_/*F*_*m*_, and reduced fresh weight under the same stress conditions [192], suggesting that increasing chloroplastic MDAR expression can improve the tolerance of tomato seedlings to certain types of environmental stress. It will be important to examine whether a similar benefit is conferred in adult plants and whether this would be reflected in fruit yield.

Overexpression of an Arabidopsis MDAR in tobacco exhibited enhanced tolerance to ozone and exhibited a reduction in H_2_O_2_ and increased photosynthetic activity when exposed to salt [191]. An increase in Asc content was observed in tobacco roots in which an Arabidopsis cytosolic MDAR was overexpressed when grown under normal conditions but not during growth in the presence of aluminum whereas DHAR-overexpressing plants maintained a larger Asc pool size prior to and following Al treatment [183]. Moreover, no difference root growth or in the degree of DNA damage was observed between MDAR-overexpressing and wild-type plants [183], suggesting that overexpression of DHAR, but not MDAR, was important in maintaining the higher level of Asc required to promote root growth in the presence of Al.

## 10. Conclusions

In contrast to the single pathway responsible for Asc biosynthesis in animals, multiple Asc biosynthetic pathways are present in plants, perhaps reflecting the importance of this molecule to plant health. The rise in atmospheric oxygen during Earth's past history would have presented a particular challenge to land-based organisms, resulting in a greater reliance on antioxidants to limit the harmful consequences resulting from increased exposure to oxygen. All of the enzymes of the Smirnoff-Wheeler Asc biosynthetic pathway are present in algae [204], demonstrating the evolution of this pathway prior to the appearance of land plants. However, as an uncharged molecule that is relatively long-lived, H_2_O_2_ can freely pass through membranes, and so diffusion from algae to the aqueous environment may provide another means to reduce their oxidative load. As this avenue is not available to land plants, ascorbic acid, along with other antioxidants, likely facilitated their colonization of land. From its role in regulating photosynthesis and as an antioxidant detoxifying exogenous and endogenously-generated ROS, to its role in regulating cell division and flowering, to its function as an enzyme cofactor in multiple enzymatic reactions, ascorbic acid has become essential to many aspects of plant growth and response programs. Vitamin C has also become vital to plants on a daily basis as it is unlikely that a plant could tolerate a single day of exposure to sunlight without ascorbic acid being used to detoxify photosynthetically generated ROS. Despite the importance of its role in detoxifying ROS, ascorbic acid is now so integrated into plant growth and development that its importance cannot be underestimated. Moreover, changes in ascorbic acid levels substantially alter the plant gene expression profile, particularly the expression of those genes associated with photosynthetic functioning [205], raising the possibility of multiple unintended consequences following alterations to Asc content. Although the effects of changes in Asc content on Calvin cycle enzyme activity have not been examined, the extent to which Asc supports photosynthetic functioning may affect the establishment of the light-dependent transthylakoid membrane pH gradient which is required for the activation of Calvin cycle enzymes. Additional roles of ascorbic acid during plant growth and in response programs that are presently unknown (e.g., nitrosative stress) also need to be identified for a full appreciation of this multifunctional molecule. Because of the complexity of its many roles, any attempts to engineer changes in ascorbic acid content in plants that improves one aspect, such as nutritional content, will require close examination of how such changes might impact the overall health and performance of the plant under field conditions. This will undoubtedly require highly targeted approaches to alter ascorbic acid content in specific cell types or tissues to achieve a desired end while limiting possible unintended consequences in other aspects of growth, development, and responses to biotic and abiotic stresses.

## Figures and Tables

**Table 1:** Approaches to increase ascorbic acid content through increasing ascorbate biosynthesis.

Species	Enzyme	Tissue	Gene source	Fold increase in Asc	Comments	Reference
Tobacco	l-Galactose dehydrogenase	Leaves	Arabidopsis	No change	Antisense suppression resulted in lower Asc	[37]
Tobacco	GDP-l-galactose phosphorylase	Leaves	Kiwifruit	3	Transient overexpression by agroinfection	[29]
Arabidopsis	GDP-l-galactose phosphorylase	Leaves	Kiwifruit	4	Stable transformant	[29]
Tobacco	GDP- l-galactose phosphorylase and GDP-mannose-3′,5′-epimerase	Leaves	Kiwifruit	7	Transient overexpression by agroinfection	[29]
Arabidopsis	d-Galacturonic acid reductase	Leaves	Strawberry	2 to 3	Enzyme is from the d-galacturonate pathway	[35]
Tobacco	l-Gulono-1,4-lactone oxidase	Leaves	Rat	7	No clear evidence that the animal Asc biosynthetic pathway exists in plants	[38]
Lettuce	l-gulono-1,4-lactone oxidase	Leaves	Rat	4 to 7	No clear evidence that the animal Asc biosynthetic pathway exists in plants	[38]
Arabidopsis	*myo*-inositol oxygenase	Leaves	Arabidopsis	2 to 3	*myo*-inositol/d-glucuronate pathway	[39]

**Table 2:** Approaches to increase ascorbic acid content or redox state through increasing DHAR expression.

Species	Tissue	Subcellular location	Gene source	Fold increase in Asc	Fold increase in Asc redox state	Consequence of increasing DHAR expression	Reference
Tobacco	Leaves	Cytosol	Wheat	2.2 to 3.9	2 to 3	Increased GSH content and redox state; increased ozone tolerance; reduced ROS; reduced photoinhibition; embryo twinning	[11, 141, 146, 162, 178, 181]
Tobacco	Leaves	Cytosol	Arabidopsis	1.9 to 2.1	2.4 to 2.6	Enhanced tolerance to ozone, drought, and salt	[182]
Tobacco	Leaves	Cytosol	Arabidopsis	1.3	1.6	Enhanced tolerance to aluminum	[183]
Tobacco	Leaves	Chloroplast	Human	No change	2	Increased GR activity; lower GSH redox state; enhanced tolerance to low temperature and oxidative stress	[179, 180]
Tobacco	Leaves	Chloroplast	Rice	1.6	2.4 to 3	Increased GSH content; decreased GSH redox state; enhanced tolerance to salt and cold stress	[184]
Potato	Leaves	Cytosol	Sesame	1.5	Not reported	1.6-fold increase in Asc in tubers as well using 35S promoter	[185]
Potato	Leaves	Cytosol	Potato	1.6	1.6	1.2-fold increase in Asc in tubers as well using 35S promoter	[186]
Potato	Leaves	Chloroplast	Potato	1.4 to 1.5	1.4 to 1.5	No increase in Asc in tubers	[186]
Arabidopsis	Leaves	Cytosol	Arabidopsis	2 to 4.25	3 to 16	Increased GSH content and redox state; enhanced tolerance to high-light and high-temperature stress	[187]
Arabidopsis	Leaves	Cytosol	Rice	1.1 to 1.4	0.9 to 1.1	Enhanced tolerance to salt stress	[188]
Maize	Leaves	Cytosol	Wheat	1.8	1.3 to 1.4	Increased GSH content and redox state	[11]
Tomato	Fruit	Cytosol	Tomato	1.6	Not reported	No increase in foliar Asc content	[189]
Potato	Tubers	Cytosol	Sesame	1.1 to 1.3	Not reported	No change in foliar Asc content using the tuber-specific patatin promoter	[185]
Maize	Kernels	Cytosol	Wheat	1.9	1 to 4	Improved nutritive value of maize grain; increased GSH content and redox state	[11]
Maize	Kernels	Cytosol	Wheat	6	Not reported	Improved nutritive value of maize grain	[13]

**Table 3:** Approaches to increase ascorbic acid content or redox state through increasing MDAR expression.

Species	Tissue	Subcellular location	Gene source	Fold increase in Asc	Fold increase in Asc redox state	Consequence of increasing MDAR expression	Reference
Tobacco	Leaves	Cytosol	Arabidopsis	1.2	1.3	No change in aluminum tolerance	[183]
Tobacco	Leaves	Cytosol	Arabidopsis	2.2	2.2 to 3	Enhanced tolerance to ozone and salt stress	[191]
Arabidopsis	Leaves	Chloroplast	Tomato	1.2	2.2	Enhanced tolerance to low- and high-temperature stress; enhanced tolerance to oxidative stress	[192]
Tomato	Fruit	Cytosol	Tomato	No change	Not reported	No increase in Asc content in leaves or green fruit	[189]

## Abbreviations

ABA: Abscisic acid
AFRR: Ascorbate-free radical reductase
AO: Ascorbate oxidase
APX: Ascorbate peroxidase
Asc: Ascorbate
CaMV: Cauliflower mosaic virus
CAT: Catalase
Chl: Chlorophyll
DHA: Dehydroascorbate
DHAR: Dehydroascorbate reductase
ETR: Electron transport rate
Fd: Ferredoxin
GalLDH: l-Galactono-1,4-lactone dehydrogenase
GulLO: l-Gulono-1,4-lactone oxidase
GalUR: d-Galacturonic acid reductase
GR: Glutathione reductase
GSH: Glutathione
GST: Glutathione-S-transferase
HRGP: Hydroxyproline-rich glycoprotein
MDA: Monodehydroascorbate reductase
MDAR: Monodehydroascorbate reductase
NAT: Nucleobase-ascorbate transporter
NPQ: Nonphotochemical quenching
*ϕ*PSII: Quantum yield of PSII
qE: Energy-dependent NPQ
qI: Photoinhibition
RbcL: Ribulose bisphosphate carboxylase/oxygenase large subunit
QC: Quiescent center
PSI: Photosystem I
PSII: Photosystem II
RDA: Recommended dietary allowance
ROS: reactive oxygen species
SOD: superoxide dismutase
TBARS: thiobarbituric acid reactive substance
VDE: violaxanthin de-epoxidase.
